# Genetic and biochemical approaches towards unravelling the degradation of gallotannins by *Streptococcus gallolyticus*

**DOI:** 10.1186/s12934-014-0154-8

**Published:** 2014-10-31

**Authors:** Natalia Jiménez, Inés Reverón, María Esteban-Torres, Félix López de Felipe, Blanca de las Rivas, Rosario Muñoz

**Affiliations:** Laboratorio de Biotecnología Bacteriana, Instituto de Ciencia y Tecnología de Alimentos y Nutrición, ICTAN-CSIC, Juan de la Cierva 3, Madrid Spain

**Keywords:** *Streptococcus gallolyticus*, Tannase, Gallate decarboxylase, Hydrolase, Gallotannins

## Abstract

**Background:**

Herbivores have developed mechanisms to overcome adverse effects of dietary tannins through the presence of tannin-resistant bacteria. Tannin degradation is an unusual characteristic among bacteria. *Streptococcus gallolyticus* is a common tannin-degrader inhabitant of the gut of herbivores where plant tannins are abundant. The biochemical pathway for tannin degradation followed by *S. gallolyticus* implies the action of tannase and gallate decarboxylase enzymes to produce pyrogallol, as final product. From these proteins, only a tannase (TanB_Sg_) has been characterized so far, remaining still unknown relevant proteins involved in the degradation of tannins.

**Results:**

In addition to TanB_Sg_, genome analysis of *S. gallolyticus* subsp. *gallolyticus* strains revealed the presence of an additional protein similar to tannases, TanA_Sg_ (GALLO_0933). Interestingly, this analysis also indicated that only *S. gallolyticus* strains belonging to the subspecies “*gallolyticus*” possessed tannase copies. This observation was confirmed by PCR on representative strains from different subspecies. In *S. gallolyticus* subsp. *gallolyticus* the genes encoding gallate decarboxylase are clustered together and close to TanB_Sg_, however, TanA_Sg_ is not located in the vicinity of other genes involved in tannin metabolism. The expression of the genes enconding gallate decarboxylase and the two tannases was induced upon methyl gallate exposure. As TanB_Sg_ has been previously characterized, in this work the tannase activity of TanA_Sg_ was demonstrated in presence of phenolic acid esters. TanA_Sg_ showed optimum activity at pH 6.0 and 37°C. As compared to the tannin-degrader *Lactobacillus plantarum* strains, *S. gallolyticus* presented several advantages for tannin degradation. Most of the *L. plantarum* strains possessed only one tannase enzyme (TanB_Lp_), whereas all the *S. gallolytcius* subsp. *gallolyticus* strains analyzed possesses both TanA_Sg_ and TanB_Sg_ proteins. More interestingly, upon methyl gallate induction, only the *tanB*_*Lp*_ gene was induced from the *L. plantarum* tannases; in contrast, both tannase genes were highly induced in *S. gallolyticus*. Finally, both *S. gallolyticus* tannase proteins presented higher activity than their *L. plantarum* counterparts.

**Conclusions:**

The specific features showed by *S. gallolyticus* subsp. *gallolyticus* in relation to tannin degradation indicated that strains from this subspecies could be considered so far the best bacterial cellular factories for tannin degradation.

**Electronic supplementary material:**

The online version of this article (doi:10.1186/s12934-014-0154-8) contains supplementary material, which is available to authorized users.

## Background

Tannins are widespread in the plant kingdom, and are often found in woody, lignified tissues [1]. Tannins form weak, pH-dependent and reversible associations with a range of substrates such as cellulose, proteins or enzymes, among others, often making the substrate resistant to microbial attack [2]. Several reports have suggested that the presence of tannins at less than 6% dry matter of the herbivore diet may result in improved animal performance. In contrast, detrimental effects of condensed tannins in excess of 6% dry matter include decreased growth rate and body weight gain, perturbation of mineral absorption, and inhibition of digestive enzymes [3]. Therefore, some herbivores have developed mechanisms to overcome adverse effects of tannins, at least partly, through the presence of tannin-resistant microorganisms [4].

*Streptococcus gallolyticus* (formerly known as *Streptococcus bovis* biotype I) has been isolated as a tannin-resistant bacterium from diverse habitats. It is a normal inhabitant of the rumen and has been isolated from feces of koalas, kangaroos, Japanese large wood mouse, cows, horses, pigs, and guinea pigs [5-7]. The presence of *S. gallolyticus* strains in the digestive tract of herbivores may play an essential role for the host in order to assimilate the tannin-rich diets from their natural habitats. The specific catabolic capacities of *S. gallolyticus* likely provide this bacterium a selective advantage to survive in the gut of herbivores, where tannins of plant origin are abundant. Therefore, a symbiotic relationship could exist between the animal host and the bacteria to counteract the antinutritional effect of dietary tannins [7].

*S. gallolyticus* strains hydrolyzed tannic acid to release gallic acid, which was subsequently decarboxylated to pyrogallol [8]. The proposed biochemical pathway for the degradation of tannins by *S. gallolyticus* implies the action of a tannase and a gallate decarboxylase enzyme to decarboxylate the gallic acid formed by tannase action [8]. Pyrogallol is formed as a final product from tannin biodegradation [8]. An identical pathway is also used by *Lactobacillus plantarum* strains to degrade tannins. The *L. plantarum* genes encoding tannase (*tanB*_*Lp*_, formerly called *tanLp1*) [9] and gallate decarboxylase (*lpdBCD*) [10] involved in tannin degradation have been identified. In addition, a second tannase gene (*tanA*_*Lp*_) present in some *L. plantarum* strains has been recently described [11]. Upon tannin induction, the expression of *tanB*_*Lp*_ was induced, whereas *tanA*_*Lp*_ expression was not affected [11]. Moreover, TanA_Lp_ has a specific activity ten times lower than the specific activity calculated for TanB_Lp_ tannase.

Similarly to *L. plantarum*, the genome of *S. gallolyticus* UCN34 revealed unique features among streptococci related to its adaptation to the rumen environment such as its ability to degrade tannins [12]. Tannins must be degraded by the action of a tannase enzyme [13]. A gene encoding a nonsecreted protein similar to TanB_Lp_ (GALLO_1609) was found in the *S. gallolyticus* UCN34 genome. This protein TanB_Sg_ (formerly called TanSg1) has been biochemically characterized recently [14]. In addition, another gene, GALLO_1609, encoding a 596-amino acid long protein 43% identical to the tannase from *Staphylococcus lugdunensis* (TanA_Sl_) [15] is present in the *S. gallolyticus* UCN34 genome. From the genes involved in tannin degradation in *S. gallolyticus*, only the *tanB*_*Sg*_ gene encoding a tannase has been identified so far [14], remaining unknown the genes encoding the gallate decarboxylase enzyme as well as a putative second tannase enzyme. In this work, *S. gallolyticus* tannase and gallate decarboxylase encoding genes involved in tannin degradation have been identified and their expression comparatively studied. In addition a novel tannase has been characterized. These results provide new relevant insights into *S. gallolyticus* tannin degradation, a rare biochemical property among bacteria.

## Results and discussion

### Sequence analysis of *S. gallolyticus* tannase enzymes

**Figure 1:**
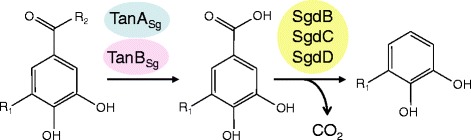
**Schematic representation of tannin degradation pathway followed by** ***S. gallolyticus*** **.** When R_1_ is (H) the represented compounds are a protocatechuate ester, protocatechuic acid, and catechol. When R_1_ is (OH) the represented compounds are a gallate ester, gallic acid, and pyrogallol. R_2_ could be (OCH_3_) representing methyl ester of gallic/protocatechuic acid, (OCH_2_CH_3_) representing ethyl esters, (OCH_2_CH_2_CH_3_) propyl esters, (glucose) as in tannic acid, or (epigallocatechin) in epigallocatechin gallate. TanA_Sg_ and TanB_Sg_ represented tannase enzymes and SgdB, SgdC, and SgdD, represent the three subunits of gallate decarboxylase enzyme.

The formerly *Streptococcus bovis* group is a large bacterial complex including different species frequently isolated from animals. In 2003, the physiological differentiation between species related to the complex and the clarification of their respective phylogenetic position was improved [16]. The updated classification included three subspecies: S. *gallolyticus* subsps. *gallolyticus*, *S. gallolyticus* subsp. *pasteurianus*, and *S. gallolyticus* subsp. *macedonicus*. These can be identified according to differential biochemical reactions. Among these biochemical tests, tannase activity was only described in *S. gallolyticus* subsp. *gallolyticus* strains. Only strains from this subspecies hydrolyze methyl gallate (tannase activity) and decarboxylate gallic acid to pyrogallol [16] (Figure 1).

The ability to degrade tannins is a specific attribute of *S. gallolyticus* subsp. *gallolyticus* deemed important in hostile environments, as tannins are toxic polyphenolic compounds that form strong complexes with proteins and other macromolecules. The *S. gallolyticus* subsp. *gallolyticus* UCN34 genome revealed the presence of two proteins similar to tannases, TanA_Sg_ (GALLO_0933) and TanB_Sb_ (GALLO_1609). The tannase proteins from *S. lugdunensis* (TanA_Sl_), *L. plantarum* (TanA_Lp_ and TanB_Lp_) and *S. gallolyticus* (TanA_Sg_ and TanB_Sg_) were aligned. The alignment revealed that two protein groups could be easily identified. TanA proteins, higher than 60 kDa and possessing a cleavage site of a peptide signal, and TanB proteins, having around 50 kDa of molecular size. The identity degree among TanA and TanB proteins is lower than 30%. The comparison of the amino acid sequence of these tannase proteins with TanB_Lp_, whose tridimensional structure have been recently solved [17], revealed that the residues important for activity are conserved. All the analyzed proteins possessed the conserved motif Gly-X-Ser-X-Gly typical of serine hydrolases. The catalytic triad identified in the TanB_Lp_ structure (Ser-163, Asp-419, and His-451) is only conserved in the TanB proteins. In both TanA proteins Asp-419 is substituted by a Gln residue. This amino acid variation was noticed previously and suggested that the conserved residue Asp-421 may fulfil the role of Asp-419 as the acidic residue of the catalytic triad [17]. The residues identified that make contacts with the three hydroxyl groups of gallic acid (Asp-421, Lys-343, and Glu-357, in TanB_Lp_) are conserved in all the tannase proteins analyzed (Additional file 1: Figure S1A). When TanA proteins were compared identity degrees ranging from 44-50% were found (Additional file 1: Figure S1B). However, the identity among TanB proteins was more lower (32%) (Additional file 1: Figure S1C).

**Figure 2:**
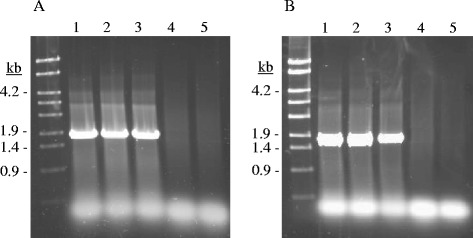
**PCR amplification of tannase encoding genes from several** ***S. gallolyticus*** **strains. (A)** Amplification of 1.7-kb DNA fragment of *tanA*_*Sg*_ with oligonucleotides 803-804. **(B)** Amplification of 1.4-kb *tanB*_*Sg*_ fragment with oligonucleotides 774-775. Chromosomal DNA from the following *S. gallolyticus* strains was used for PCR amplification: *S. gallolyticus* DSM 13808 (1), *S. gallolyticus* subsp. *gallolyticus* DSM 16831^T^ (2), *S. gallolyticus* subsp. *gallolyticus* UCN34 (3), *S. gallolyticus* subsp. *pasteurianus* DSM 15351^T^ (4), and *S. gallolyticus* subsp. *macedonius* DSM 15879^T^ (5). PCR fragments were subject to agarose gel electrophoresis and stained with Gel Red. Left lane, λ-EcoT14I digest (Takara). Numbers indicated some of the molecular sizes (in kb).

As described previously for *S. gallolyticus* spp*.*, only strains from the subspecies *gallolyticus* possess tannase activity [16]. However, it is not known if both tannase proteins are present in all the *S. gallolyticus* subsp. *gallolyticus* strains. Currently, the genomes of four *S. gallolyticus* subsp. *gallolyticus* strains are available (UCN34, ATCC 43143, ATCC BAA-2069, and TX2005 strains). A genome search revealed that the four strains possessed both tannase genes, TanA_Sg_ (GALLO_0933, SGGB_0917, SGGBAA2069_c09070/80, and HMPREF9352_1611) or TanB_Sg_ (GALLO_1609, SGGB_1624, SGGBAA2069_c16370, and HMPREF9352_0937) in UCN34, ATCC 43143, ATCC BAA-2069, and TX2005 strains, respectively. In relation to TanA_Sg_ from UCN34 strain, the proteins from TX2005 and ATCC 43143 strains exhibited 3 or 5 amino acid changes (data not shown). In addition, the TanA_Sg_ protein from ATCC BAA-2069 posses a mutation at position Tyr-230 which originates a stop codon, and therefore, a truncated protein (data not shown). In relation to TanB_Sg_ proteins, protein from UCN34, ATCC 43143, and ATCC BBA-2069 strains were identical, however, protein from TX2005 strain showed 20 amino acid substitutions (data not shown). These results seem to indicate that all the strains from the *S. gallolyticus* subsp. *gallolyticus* possessed both, *tanA*_*Sg*_ and *tanB*_*Sg*_ genes. In order to associate the presence of these genes with the “*gallolyticus*” subspecies, DNA from different *S. gallolyticus* subspecies was used to amplify *tanA*_*Sg*_ and *tanB*_*Sg*_ genes. Oligonucleotides 803-804, and 774-775 amplified 1.7 and 1.4 kb DNA fragments from *tanA*_*Sg*_ and *tanB*_*Sg*_, respectively. From the strains assayed, only *S. gallolyticus* DSM 13808, *S. gallolyticus* subsp. *gallolyticus* UCN34, and *S. gallolyticus* subsp. *gallolyticus* DSM 16831^T^ strains produce the two amplicons (Figure 2). No amplification was observed in strains belonging to the other *S. gallolyticus* subspecies, e.g. *S. gallolyticus* subsp. *pasteurianus* DSM 15351^T^, and *S. gallolyticus* subsp. *macedonius* DSM 15879^T^. These results indicated that, similarly to tannase activity, the presence of *tanA*_*Sg*_ and *tanB*_*Sg*_ genes seems to be specific for the subspecies *gallolyticus*.

### Tannin-induced gene expression of the *S. gallolyticus* region involved in tannin degradation

**Figure 3:**
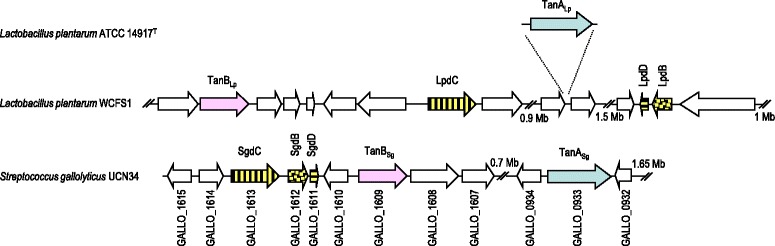
**Genetic organization of the** ***S. gallolyticus*** **subsp.** ***gallolyticus*** **UCN34 chromosomal region containing gallate decarboxylase and tannase encoding genes (accession NC_013798, positions 1708280/c-1698323/c and 984535/c-981288/c).** The genetic organization of the chromosomal region containing the same genes in *L. plantarum* WCFS1 (NC_004567) or ATCC 14917^T^ (ACGZ02000013.1) is also represented. Arrows indicate genes. Genes coding for TanA tannase proteins are coloured in blue and TanB in pink. Genes encoding gallate decarboxylase subunits are coloured in yellow and each different subunit is represented by different drawing.

The presence of both tannase genes in all the *S. gallolyticus* subsp. *gallolyticus* strains analyzed is in contrast with the uncommon presence of *tanA*_*Lp*_ among *L. plantarum* strains. Only 4 strains out of 29 *L. plantarum* strains analyzed possessed both tannase genes. Another significant difference among *L. plantarum* and *S. gallolyticus* strains is the organization of the genes involved in tannin degradation. In *L. plantarum* the genes encoding gallate decarboxylase (*lpdC*, and *lpdBD*) are separated in the chromosome by more than 1 Mb [10]; however in *S. gallolyticus*, the genes encoding gallate decarboxylase are clustered together (*sgdCBD* or GALLO_1613, GALLO_1612, and GALLO_1611, respectively) (Figure 3). Regarding the region involved in tannin degradation, all the currently available *S. gallolyticus* genomes displayed the same genetic organization as the *S. gallolyticus* UCN34 strain (Figure 3). In *S. gallolyticus* strains, TanA_Sg_ and TanB_Sg_ are separated from 691 (TX2005 strain) to 750 kb (ATCC BAA-2069 strain). Similarly to *L. plantarum*, TanB is located close to subunit C, the catalytic subunit of the gallate decarboxylase. However, TanA is not located in the vicinity of other genes involved in the metabolism of tannins in *L. plantarum* and *S. gallolyticus*. The different chromosomal location of both tannase genes could indicate a different function and regulation.

**Figure 4:**
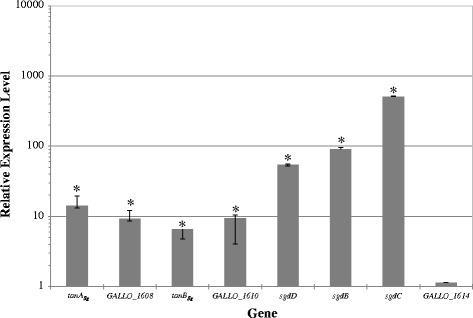
**Comparison of the relative expression levels of tannase and gallate decarboxylase genes in response to methyl gallate exposure.** Relative expression levels in 15 mM methyl gallate were calculated with the 7500 Fast System relative quantification software using *S. gallolyticus 16S rRNA* gene as endogenous gene and the growth in the absence of methyl gallate as growth condition calibrator. The experiments were done in triplicate. The mean value and the standard error are shown. Asteriks indicate a *p* value <0.1.

In order to know the specific physiological role of both *S. gallolyticus* tannases the study of their synthesis under the presence of a substrate could provide relevant data. As tannase and gallate decarboxylase are involved in tannin degradation, the relative expression of their encoding-genes under methyl gallate exposure was studied. *S. gallolyticus* UCN34 cultures were induced for 10 min by the presence of 15 mM methyl gallate as potential tannase substrate. Figure 4 shows the relative expression levels of *tanA*_*Sg*_, *tanB*_*Sg*_, and *sgdCBD* genes under methyl gallate exposure. The highest induction was observed in the genes encoding gallate decarboxylase, *sgdC*, *sgdB* and *sgdD* which increased their expression 507, 90 and 54 times, respectively. Similarly, *tanA*_*Sg*_ and *tanB*_*Sg*_ genes were induced 14- and 6-fold, respectively. This result is in contrast with the expression pattern observed on the equivalent tannase proteins from *L. plantarum* since the presence of 15 mM methyl gallate slightly induces (about 3-fold) the expression of *tanB*_*Lp*_ gene. The expression behaviour observed in *S. gallolyticus* UCN34 allow us to assume that, in the presence of substrate, all the genes involved in tannin degradation were highly induced.

### Biochemical properties of *S. gallolyticus* UCN34 TanA_Sg_ tannase

When TanB_Lp_ was the only tannase described from *L. plantarum*, homology searched allowed us to identify *tanB*_*Sg*_ (GALLO_1609). The gene was cloned and expressed in *E. coli*, and the recombinant TanB_Sg_ protein was biochemically characterized [14]. The data indicated that, as compared to TanB_Lp_, TanB_Sg_ possesses remarkable biochemical properties. TanB_Sg_ has a specific activity 41% higher than TanB_Lp_, and displays optimum activity at pH 6-8 and 50-70°C, showing high stability over a broad range of temperatures [14]. However, in relation to its substrate range and contrarily to TanB_Lp_, only esters with a short aliphatic alcohol were effectively hydrolyzed by TanB_Sg_. Taking into account only the activity of TanB tannases, it seems that *S. gallolyticus* could hydrolyze tannins more efficiently than *L. plantarum* strains.

**Figure 5:**
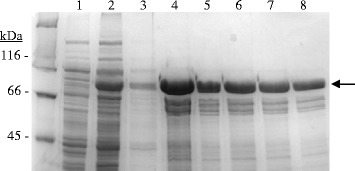
**SDS-PAGE analysis of the expression and purification of** ***S. gallolyticus*** **TanA**_**Sg**_ **protein.** Analysis of soluble cell extracts of IPTG-induced *E. coli* BL21(DE3) (pURI3-TEV) (1) or *E. coli* BL21(DE3) (pURI3-TEV-TanA_Sg_) (2), flowtrough (3), or fractions eluted after His affinity resin (4-8). The arrow indicated the overproduced and purified protein. The gel was stained with Coomassie blue. Molecular mass markers are located at the left (SDS-PAGE Standards, Bio-Rad).

Recently it has been described that in a few *L. plantarum* strains, a second tannase gene (*tanA*_*L*p_) could be found [11]. Compared to TanB_Lp_, the TanA_Lp_ protein presented ten times lower specific activity, and showed differences in optimal pH and temperature. In order to demonstrate the functionality of TanA_Sg_ protein, and to know its biochemical properties and relevance on tannin degradation, the *tanA*_*Sg*_ gene was expressed in *E. coli* under the control of an IPTG inducible promoter. Cell extracts were used to detect the presence of overproduced proteins by SDS-PAGE analysis. Whereas control cells containing the pURI3-TEV vector did not show protein overexpression, an overproduced protein with a molecular mass around 63 kDa was apparent with cells harbouring pURI3-TEV-TanA_Sg_ (Figure 5). Since the cloning strategy yields a His-tagged protein variant, *S. gallolyticus* pURI3-TEV-TanA_Sg_ could be purified on an immobilized metal affinity chromatography (IMAC) resin. The recombinant protein was eluted from the resin at 150 mM imidazole, and observed as single band on 10% SDS-PAGE (Figure 5).

**Figure 6:**
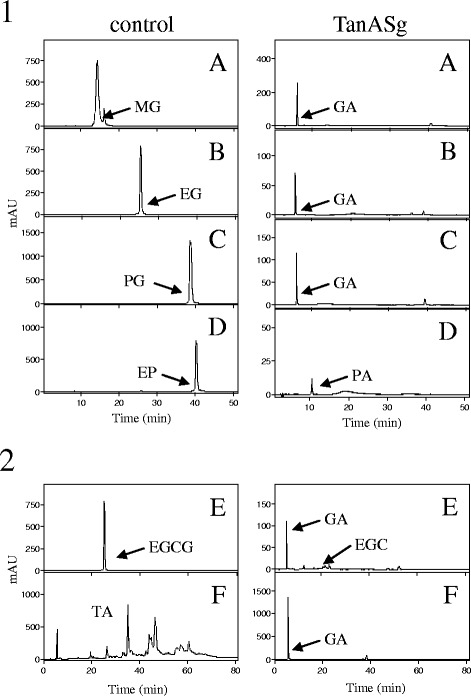
**Tannase activity of** ***S. gallolyticus*** **TanA**_**Sg**_ **protein against simple phenolic esters (1) and complex tannins (2).** Hydrolase activity of purified TanA_Sg_ protein was analyzed on triplicate and compared with control reactions on which the enzyme was omitted. HPLC chromatograms of TanA_Sg_ (100 μg) incubated in 1 mM of methyl gallate **(A)**, ethyl gallate **(B)**, propyl gallate **(C)**, ethyl protocatechuate **(D)**, epigallocatechin gallate **(E)**, and tannic acid **(F)**. The methyl gallate (MG), ethyl gallate (EG), propyl gallate (PG), ethyl protocatechuate (EP), epigallocatechin gallate (EGCG), tannic acid (TA), gallic acid (GA), protocatechuic acid (PA), and epigallocatechin (EGC) detected are indicated. The chromatograms were recorded at 280 nm.

To demonstrate tannase activity, the TanA_Sg_ protein purified by affinity chromatography was incubated in the presence of different esters of gallic acid. As expected for a tannase enzyme, TanA_Sg_ was able to hydolyze esters from gallic acid, confirming their tannase activity (Figure 6-1). TanA_Sg_ hydrolyzes methyl, ethyl and propyl gallate, but similarly to TanB_Sg_ [14] and TanA_Lp_ [11], was unable to hydrolyze esters having an alcohol substituent as long as lauryl, which was hydrolyzed by TanB_Lp_ [18]. As a general rule of the bacterial tannases described so far, a protocatechuate ester (ethyl protocatechuate) was also hydrolyzed by TanA_Sg_ [18]. Esters from other phenolic acids assayed were not hydrolyzed (data not shown). These results confirmed that TanA_Sg_ is a functional tannase with a substrate range resembling the bacterial tannases described previously [11,14,18].

Different dietary plant varieties produce different types and quantities of phenolic compounds [19]. As the digestive tract of herbivores probably contains different tannins, we focused our studies on the relatively well-define commercially available preparations, tannic acid and epigallocatechin gallate. Tannic acid is almost exclusively formed by poly-galloyl glucose, and gallic acid was observed as the final product of TanA_Sg_ activity on tannic acid (Figure 6-2). Similarly, gallic acid was released from epigallocatechin as a result of the action of TanA_Sg_ on epigallocatechin gallate. These results confirmed the capacity of TanA_Sg_ to degrade dietary tannins.

Once the tannase activity of TanA_Sg_ was confirmed, their biochemical properties were determined. Since tannase catalyzes the hydrolysis of the galloyl ester linkage releasing gallic acid, the activity of tannase could be measured by estimating the gallic acid formed by the enzyme action [20]. A rhodanine specific method for the detection of gallic acid was used for a reliable quantification of tannase activity [21]. Rhodanine reacts with the vicinal hydroxyl groups of gallic acid to give a red complex with a maximum absorbance at 520 nm. Rhodanine assay was used to determine the specific activity of TanA_Sg_. Using methyl gallate as substrate, the specific activity of TanA_Sg_ was 256 U/mg, 55% or 37% lower than the specific activity of TanB_Sg_ (577 U/mg) or TanB_Lp_ (408 U/mg); however, it was more than 6 times higher than the activity of the equivalent protein from *L. plantarum*, TanA_Lp_ (39 U/mg). These activity data indicated that *S. gallolyticus* strains have more potential to degrade tannins than *L. plantarum* strains. These activity data indicated that *S. gallolyticus* strains have more potential to degrade tannins than *L. plantarum* strains. Firstly, in *L. plantarum* only a small number of strains possessed *tanA*_*Lp*_ gene, an inducible gene, whereas all the *S. gallolytius* subsp. *gallolyticus* strains analyzed so far possesses both tannase genes, being the two genes inducible by gallate esters. More importantly, tannases from *S. gallolyticus* possess higher specific activity than their counterparts from *L. plantarum*. A roughly estimation shows that *S. gallolyticus* strains bearing TanA_Sg_ and TanB_Sg_ posses a two-fold higher tannase activity than most *L. plantarum* strains possessing only TanB_Lp_ tannase.

**Figure 7:**
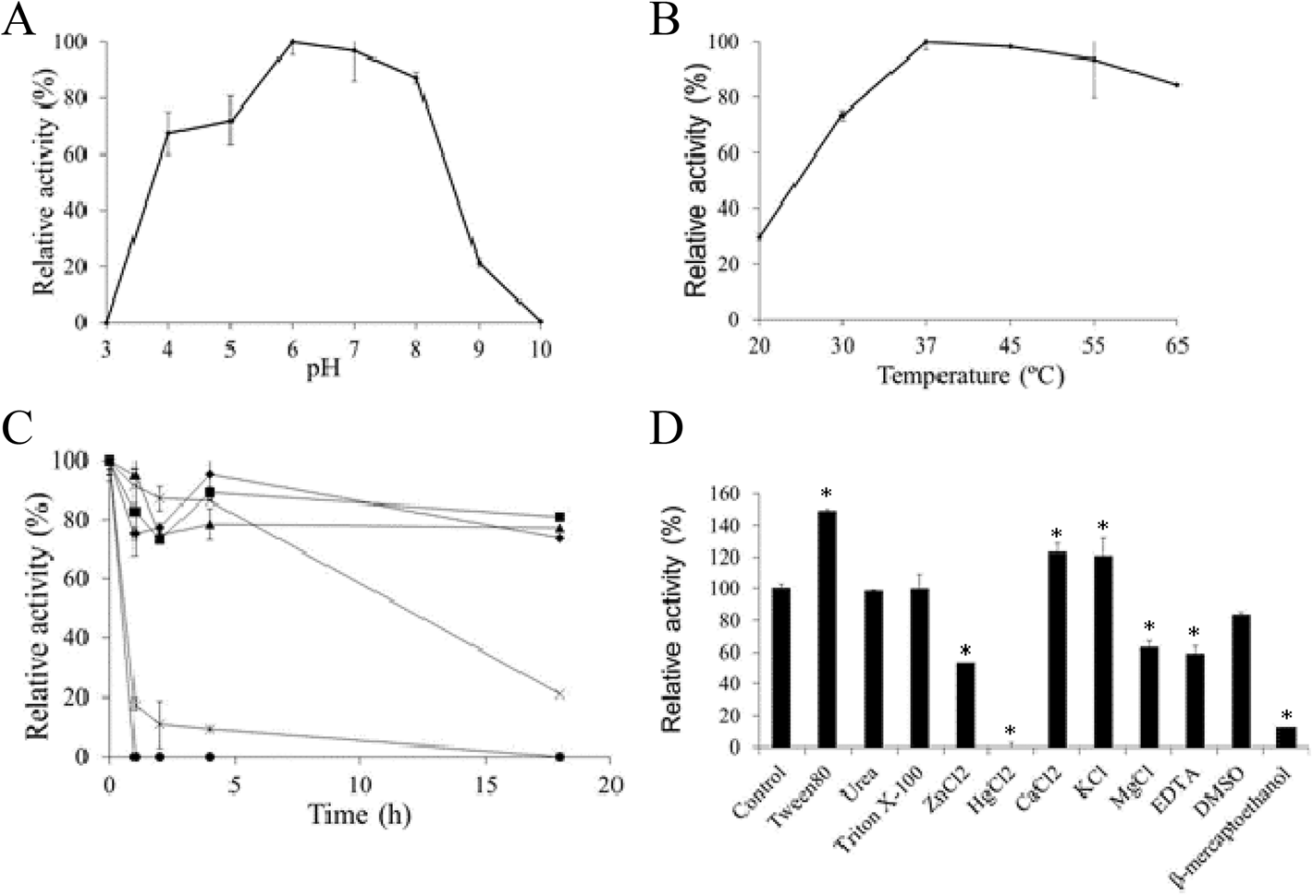
**Some biochemical properties of TanA**_**Sg**_ **protein. (A)** Relative activity versus pH. **(B)** Relative activity of TanA_Sg_ versus temperature. **(C)** Thermal stability of TanA_Sg_ after preincubation at 22°C (filled diamond), 30°C (filled square), 37°C (filled triangle), 45°C (cross), 55°C (star), and 65°C (filled circle) in phosphate buffer (50 mM, pH 6.5); at indicated times, aliquots were withdrawn, and analyzed as described in the Methods section. The observed maximum activity was defined as 100%. **(D)** Relative activity of TanA_Sg_ after incubation with 1 mM concentrations of different additives. The activity of the enzyme in the absence of additives was defined as 100%. The experiments were done in triplicate. The mean value and the standard error are shown. Asteriks indicate a *p* value <0.05.

In order to known if TanA_Sg_ possesses additional valuable biochemical features, its optimal pH and temperature was analyzed. The biochemical characterization of TanA_Sg_ revealed an optimal pH around 6, being also highly active at pH 6-8 (Figure 7A). This optimal pH is slightly lower than that reported for TanB_Lp_ (optimal pH 7.0-8.0) [9,18], but similar than that of TanB_Sg_ [14] and TanA_Lp_ [11]. The optimum temperature for activity is 37°C, however the enzyme also exhibited very high activity at higher temperatures (Figure 7B). At 65°C TanA_Sg_ showed more than 80% of the maximum activity. TanA_Sg_ is able to hydrolyze the substrate (methyl gallate) at high temperatures. In addition, TanA_Sg_ showed an improved thermal stability compared to TanB_Lp_, since it retained more than 70% of the maximal activity after 18 h incubation at 37°C (Figure 7C). Non-ionic detergents showed different effect on TanA_Sg_ activity. Whereas Triton-X-100 did not affect activity, Tween-80 greatly increased tannase activity (Figure 7D). Similarly to TanB_Lp_, among metal ions, Ca^2+^ and K^+^ increased and Hg^2+^ greatly inhibited TanA_Sg_ activity [18]. The ions Zn^2+^ and Mg^2+^ partially inhibited the enzyme. The activity of TanA_Sg_ was also significantly inhibited by β-mercaptoethanol (Additional file 2: Table S1).

In addition to TanB_Sg_ activity, the biochemical properties showed by TanA_Sg_ such as high specific activity, optimal temperature (37°C) and broad pH range indicated that *S. gallolyticus* subsp. *gallolyticu*s strains are so far the bacterial cells better adapted to degrade the tannins present on the diet. It has been described that *S. gallolyticus* initially responds to tannins with an increased lag phase and decreased growth rate [22]. The response mechanisms used by *S. gallolyticus* strains enables them to maintain growth and biosynthetic capacity in the presence of high tannin concentrations [22].

## Conclusions

In summary, this study confirmed among bacteria the specific characteristics present in *S. gallolyticus* subsp. *gallolyticus* strains which could be important for survival in the gut environment of herbivores, where a large diversity of tannins is present. *S. gallolyticus* subsp. *gallolyticus* should be able to degrade these polyphenols and it is known that this bacterium does not depend on other microorganisms for their degradation. The tannin degradation pathway followed by *S. gallolyticus* subsp. *gallolyticus* strains implies the combined action of tannase and gallate decarboxylase, which are close on *S. gallolyticus* genome and seems to act coordinately. In addition, the presence in all the *S. gallolyticus* subsp. *gallolyticus* strains of a second, inducible and extracellular tannase enzyme provide them additional advantages for the degradation of high-molecular tannins. The specific features showed by *S. gallolyticus* subsp. *gallolyticus* in relation to tannin degradation suggested that these strains are the best bacterial factories for tannin degradation described so far. This explains the widespread occurrence of *S. gallolyticus* in the rumen of livestock that frequently browse tannin-containing forages, and it is likely that the presence of *S. gallolyticus* provides a selective advantage to these animals.

## Materials and methods

### Bacterial strains and growth conditions

*S. gallolyticus* subsp. *gallolyticus* UCN34 (CIP 110142) used through this study was kindly provided by Dr. Philippe Glaser (Institut Pasteur, France). *S. gallolyticus* DSM 13808, *S. gallolyticus* subsp. *gallolyticus* DSM 16831^T^, *S. gallolyticus* subsp. *pasteurianus* DSM 15351^T^, and *S. gallolyticus* subsp. *macedonius* DSM 15879^T^ were purchased from the German Collection of Microorganisms and Cell Cultures (DSMZ). *Escherichia coli* DH10B and *E. coli* BL21 (DE3) were used as transformation and expression hosts in the pURI3-TEV vector [23]. The *S. gallolyticus* strains were grown in BHI medium at 37°C without shaking, and the *E. coli* strains were cultured in Luria-Bertani (LB) medium at 37°C and shaking at 200 rpm.

### PCR detection of tannase encoding genes

Genes encoding *S. gallolyticus* tannases (*tanA*_*Sg*_ and *tanB*_*Sg*_) were amplified by PCR using chromosomal DNA from *S. gallolyticu*s strains. The *tanA*_*Sg*_ gene (1.7 kb) was amplified by using primers 803 and 804 (Additional file 3: Table S2). Oligonucleotides 774 and 775 were used to amplify *tanB*_*Sg*_ gene (1.4 kb). The reactions were performed in a Personal thermocycler (Eppendorf), using 30 cycles of denaturation at 94°C for 30 s, annealing at 55°C for 1 min, and extension at 72°C for 2 min. Amplified fragments were resolved in agarose gels.

### Quantitative PCR

For RNA isolation, BHI cultures of *S. gallolyticus* subsp. *gallolyticus* UCN34 were grown up to an OD 600_nm_ of 1 and then supplemented with methyl gallate at 15 mM final concentration. As control, RNA was also isolated from cultures not supplemented with methyl gallate. The induced cells and their corresponding controls were centrifuged at 4°C after 10 min of exposure to the gallate ester. The pellet was mixed with 2 mL of quenching buffer (60% methanol, 66.7 mM HEPES, pH 6.5, -40°C). Following quenching, the cells were centrifuged at 9,000 × g for 10 min at -10°C and suspended in an extraction mixture (500 μL 1:4 chloroform-acid phenol, 30 μL of 10% SDS, 30 μL Na-acetate 3 M pH 5.2, 400 μL Tris-EDTA buffer [10 mM Tris(hydroxymethyl)amino methane, 1 mM EDTA] pH 7.4, 15 mg of polyvinylpoly-pyrrolidone, and 500 mg of glass beads (ϕ, 75-150 μm). The cells were broken under frozen conditions in a Mini-Bead Beater (Biospec Products, INC.) using three treatments of 4600 rpm for 40 seconds and chilled 1 min between cycles. The suspension was then centrifuged at 4°C at 10,000 × g for 2 min. After two extractions with 500 μL of chloroform the supernatant containing the RNA was immediately frozen in liquid nitrogen, and stored at -80°C. NanoDrop ND1000 instrument was used for quantification of RNA. The A_260_/A_280_ and A_260_/A_230_ ratios were measured to check RNA purity. Integrity and quality of RNA samples were determined by electrophoresis on agarose gels. Two treatments with DNase I (Ambion) were applied and the absence of genomic DNA was confirmed by PCR [24]. The DNA-free RNA was reverse transcribed using the High Capacity cDNA Reverse Transcription Kit (Applied Biosyntems) according to the manufacturer instructions. From the cDNA obtained, quantitative gene expression was analyzed in an AbiPrism 7500 Fast Real Time PCR system (Applied Biosystems). The SYBR Green method was used and each assay was performed in triplicate using SYBR Green real-time PCR Master Mix (Applied Biosystems). Amplification was initiated at 95°C for 10 min, followed by 40 cycles of 95°C for 15 s and 60°C for 1 min. Control PCRs were included to confirm the absence of primer dimer formation (no-template control), and to verify that there was no DNA contamination (without RT enzyme negative control). Specific primer pairs were designed with the Primer Express 3.0 program to amplify internal regions of tannase and gallate decarboxylase encoding genes (Additional file 3: Table S2). All qPCR assays amplified a single product as determined by melting curve analysis and by electrophoresis. A standard curve was plotted with cycle threshold (Ct) values obtained from amplification of known quantities of cDNA and used to determine the efficiency (E) as E = 10^-1/slope^. In order to measure *S. gallolyticus* gene expression, amplification of the endogenous control gene was performed simultaneously and its relative expression compared with that of the target gene. Results were analyzed using the comparative Ct method (also named double delta-delta Ct, 2^ΔΔCt^ method). Genes were considered differentially expressed when a nominal p-values were <0.1 and had a fold change (FC) ≥2. Relative expression levels were calculated with the 7500 Fast System relative quantification software using *S. gallolyticus 16S rRNA* gene as endogenous gene and the growth in the absence of methyl gallate as growth condition calibrator.

### Cloning, expression and purification of TanA_Sg_ from *S. gallolyticus* UCN34

The gene encoding a putative tannase (*GALLO_0933*, or *tanA*_*Sg*_) in *S. gallolyticus* UCN34 (accession YP_003430356) was PCR-amplified by PrimeSTAR HS DNA polymerase (Takara). As a peptide signal was predicted in the TanA_Sg_ sequence, primers 803 and 804 were used to amplify and clone TanA_Sg_ lacking the 26-amino acid peptide signal sequence. The gene was cloned into the pURI3-TEV vector which encodes expression of a leader sequence containing a six-histidine affinity tag. The purified 1.7-kb PCR product was then inserted into the vector by using a restriction enzyme- and ligation-free cloning strategy [23].

*E. coli* DH10B cells were transformed, recombinant plasmids were isolated, and those containing the correct insert were identified by restriction enzyme analysis, verified by DNA sequencing, and then transformed into *E. coli* BL21 (DE3) cells for expression. *E. coli* BL21 (DE3) cells carrying the recombinant plasmid, pURI3-TEV-TanA_Sg_, were grown at 37°C in LB media containing ampicillin (100 μg/ml) on a rotary shaker (200 rpm) until an optical density at 600 nm of 0.4 was reached. Isopropyl-β-D-thiogalactoside (IPTG) was added to a final concentration of 0.4 mM and protein induction was continued at 22°C for 18 h.

The induced cells were harvested by centrifugation (8,000 *g*, 15 min, 4°C), resuspended in phosphate buffer (50 mM, pH 6.5) and disrupted by French Press passages (three times at 1,100 psi). The insoluble fraction of the lysate was removed by centrifugation at 47,000 *g* for 30 min at 4°C. The supernatant was filtered through a 0.2 μm pore-size filter and then applied to a Talon Superflow resin (Clontech) equilibrated in phosphate buffer (50 mM, pH 6.5) containing 3 mM NaCl and 10 mM imidazole to improve the interaction specificity in the affinity chromatography step. The bound enzyme was eluted using 150 mM imidazole in the same buffer. The purity of the enzymes was determined by SDS-PAGE in Tris-glycine buffer. Fractions containing the His6-tagged protein were pooled and analyzed for tannase activity.

### Tannase activity assay

Tannase activity was determined using a rhodanine assay specific for gallic acid [21]. Rhodanine reacts only with gallic acid and not with galloyl esters or other phenolics. Gallic acid analysis in the reactions was determined using the following assay. Tannase enzyme (100 μg) in 700 μl of 50 mM phosphate buffer pH 6.5 was incubated with 40 μl of 25 mM methyl gallate (1 mM final concentration) for 5 min at 37°C. After this incubation, 150 μl of a methanolic rhodanine solution (0.667% w/v rhodanine in 100% methanol) were added to the mixture. After 5 min incubation at 30°C, 100 μl of 500 mM KOH was added. After an additional incubation of 5-10 min, the absorbance at 520 nm was measured on a spectrophotometer. A standard curve using gallic acid concentration ranging from 0.125 to 1 mM was prepared. One unit of tannase activity was defined as the amount of enzyme required to release 1 μmol of gallic acid per minute under standard reaction conditions.

### Biochemical properties of TanA_Sg_ tannase

The pH profile of TanA_Sg_ activity was determined using different 100 mM buffer systems containing acetic acid-sodium acetate (pH 3.0-5.0), citric acid-sodium citrate (pH 6), sodium phosphate (pH 7), Tris-HCl (pH 8), glycine-NaOH (pH 9), and sodium carbonate-bicarbonate (pH 10). The rhodanine assay was used for the optimal pH characterization of tannase. Since the rhodanine-gallic acid complex forms only in basic conditions, after the completion of the enzymatic degradation of methyl gallate, KOH was added to the reaction mixture to ensure that the same pH value (pH 11) was achieved in all samples assayed. Determinations were done in triplicate.

The optimal temperature for purified TanA_Sg_ tannase was determined in the temperature range 4-65°C in 25 mM phosphate buffer (pH 6.5). For determination of the thermal stability of TanA_Sg_, the enzyme in 50 mM phosphate buffer pH 6.5 was preincubated in the absence of substrate at 22, 30, 37, 45, 55 and 65°C for 30 min and 2, 4, 6, and 18 h. Aliquots were withdrawn in triplicate at these incubation times to test the remaining activity under standard conditions. The residual tannase activity was determined at 37°C. The non-heated enzyme was considered as control (100%).

The enzyme was pre-incubated in the presence of various metal salts and chemical agents using final concentrations of 1 mM. The effect of chemical inhibitors and stimulators on TanA_Sg_ activity was investigated in triplicate by the rhodanine assay using methyl gallate as substrate. The additives analyzed were MgCl_2_, KCl, CaCl_2_, HgCl_2_, ZnCl_2_, Triton X-100, Urea, Tween 80, EDTA, DMSO, and β-mercaptoethanol. The extent of inhibition or activation of tannase activity was indicated as the percentage of the ratio of residual activity to complete enzyme activity in the control sample without addition of metal ions or chemical agents. Tannase activity measured in the absence of any additive was used as control (100%).

The substrate specificity of TanA_Sg_ was determined using 17 commercial phenolic esters (methyl gallate, ethyl gallate, propyl gallate, lauryl gallate, methyl benzoate, ethyl benzoate, methyl 4-hydroxybenzoate, ethyl 4-hydroxybenzoate, propyl 4-hydroxybenzoate, butyl 4-hydroxybenzoate, methyl vanillate, methyl 2, 4-dihydroxybenzoate, methyl gentisate, methyl salicylate, ethyl 3, 4-dihydroxybenzoate, ferulic methyl ester, and ferulic ethyl ester) as well as a natural tannins (epigallocatechin gallate, and tannic acid). As controls, phosphate buffer (50 mM, pH 7.0) containing thereagents but lacking the enzyme were incubated in the same conditions.

The hydrolysis products were extracted twice with ethyl acetate (Lab-Scan, Ireland) and analyzed by HPLC-DAD. A Thermo (Thermo Electron Corporation, Waltham, Massachussetts, USA) chromatograph equipped with a P4000 SpectraSystem pump, and AS3000 autosampler, and a UV6000LP photodiode array detector were used. A gradient of solvent A (water/acetic acid, 98:2, v/v) and solvent B (water/acetonitrile/acetic acid, 78:20:2, v/v/v) was applied to a reversed-phase Nova-pack C_18_ (25 cm × 4.0 mm i.d.) 4.6 μm particle size, cartridge at room temperature as follows: 0-55 min, 80% B linear, 1.1 ml/min; 55-57 min, 90% B linear, 1.2 ml/min; 57-70 min, 90% B isocratic, 1.2 ml/min; 70-80 min, 95% B linear, 1.2 ml/min; 80-90 min, 100% linear, 1.2 ml/min; 100-120 min, washing 1.0 ml/min, and reequilibration of the column under initial gradient conditions. Detection was performed by scanning from 220 to 380 nm. Samples were injected onto the cartridge after being filtered through a 0.45 μm PVDF filter.

### Statistical analyses

Two-tailed Student’s test performed using GraphPad InStat, version 3.0 (GraphPad Software, San Diego, CA) was used to determine the differences between means. The data are representative means of at least three independent experiments.

## Additional files

Additional file 1: Figure S1. Comparison of amino acid sequences of bacterial tannases. **(A)** TanA_Sl_ from *Staphylococcus lugdunensis*, TanA_Lp_ and TanB_Lp_ from *Lactobacillus plantarum*, and TanA_Sg_ and TanB_Sg_ from *Streptococcus gallolyticus* subsp. *gallolyticus*. **(B)** Alignment of TanA or **(C)** TanB proteins. Multiple alignments were done using the program ClustalW2 after retrieval of sequences from BLAST homology searches. Residues that are identical (*), conserved (:) or semiconserved (.) in all sequences are indicated. Dashes indicated gaps introduced to maximize similarities. The vertical line indicated the predicted peptide signal cleavage site. The serine hydrolase conserved motif is highlighted in yellow; residues of the catalytic triad identified in the structure of TanB_Lp_ are highlighted in blue; and residues which make contacts with the three hydroxyl groups of gallic acid are highlighted in pink color. Additional file 2: Table S1. Characteristics of *S. gallolyticus* tannases. Additional file 3: Table S2. Primers used for PCR and qPCR analysis.
