# Supplementary material for: Genetic and biochemical approaches towards unravelling the degradation of gallotannins by Streptococcus gallolyticus
Source: Microb Cell Fact. 2014 Oct 31;13:154. doi: 10.1186/s12934-014-0154-8 (PMC4218992; doi:10.1186/s12934-014-0154-8)
Supplement: Additional file 1: Figure S1. — Comparison of amino acid sequences of bacterial tannases. (A) TanASl from Staphylococcus lugdunensis, TanALp and TanBLp from Lactobacillus plantarum, and TanASg and TanBSg from Streptococcus gallolyticus subsp. gallolyticus. (B) Alignment of TanA or (C) TanB proteins. Multiple alignments were done using the program ClustalW2 after retrieval of sequences from BLAST homology searches. Residues that are identical (*), conserved (:) or semiconserved (.) in all sequences are indicated. Dashes indicated gaps introduced to maximize similarities. The vertical line indicated the predicted peptide signal cleavage site. The serine hydrolase conserved motif is highlighted in yellow; residues of the catalytic triad identified in the structure of TanBLp are highlighted in blue; and residues which make contacts with the three hydroxyl groups of gallic acid are highlighted in pink color. [file 12934_2014_154_MOESM1_ESM.doc]

Figure S1

**A**

TanASl -MKKTFISLLS----ATVILSGCGVGEHQNNNSNHDAKGVN--TSNVKIKNYNQASSALQ 53

TanALp MQFRKIVPLMSGLLVMSVGLAACGHSETKTKHPTSTVAKVAKATKQTVTKADVKNAKKLL 60

TanASg MPRKKWFFTSSAVLLCSAMLLTACSSSSNSSTSSSSSQNTT--ASTSSLSSGEVSTTLDK 58

TanBLp ------------------------------------------------------------

TanBSg ------------------------------------------------------------

TanASl IDNSKWKYDSKNNVYYQLNISYVSNPQAKNVEKLGIYVPAAYFKGKKNHNGTYTVTVNDA 113

TanALp INQKQWHYNATNKVYYQVGVKYGTKTTSSTYESMGIFIPAKYVNAKASGQKTYTITFNNK 120

TanASg VDNSKWQYNADDNVYYQIGISYAANPTDAEQQTLSIFVPGDYMTATDNGNGTYTCEINTS 118

TanBLp -MSNRLIFDADWLVPEQVQVAGQAIQYYAARNIQYVQHPVAAIQVLN---VFVPAAYLHG 56

TanBSg MSINQWIFDETNNCYMSLKNVYCAQPKDSELEALHIFVPAVYMTADG---TIDRDAVVTN 57

.: :: .: : : : * .

TanASl KKVNGYSARTAPIVYPVNTPGYAEQSAPT------SYRYSNISKYMKAGFIYVEAGLRGR 167

TanALp AKVKGYTAKTAPIVMPVNTPGYAAQTAPT------GYDSS-ANKYTKAGFIYVAAGCRGL 173

TanASg ATVGNYTSETAPIVIPINTPGYSAMSALT------EYTSD-ATDYTSQGMIYVSAGLRGR 171

TanBLp SSVNGYQRATAPILMPNTVGGYLPGPADDPQRVTWPTNAGTIQQALKRGYVVVAAGIRGR 116

TanBSg KNGTIYTSQTVPIIFYNDIGGYAECQP--------AMVTPRNQRYLEDGYVLVSVGARGR 109

. * *.**: ** . . * : * .* **

TanASl SMSMGNNSSNASTKSYETGSPWGVTDLKAAIRYYRFNDSSLPGNSSKIYTFGHSGGGAQS 227

TanALp SQSDKSNGS----------SPWGVTDLKAAVRTLRLNRSRIAGNTNRVFTFGHSGGGAQS 223

TanASg DSG----------------APSGVTDAKAAIRYLRYNQGNISGNTDSIFVFGMSGGGAQS 215

TanBLp TTVDKSGQRVG-------QAPAFIVDMKAAIRYVKYNQGRLPGDANRIITNGTSAGGATS 169

TanBSg ----QSQNGIG-------KAPAGLVDLKAAVRWLRKHHNDIPGDIEKIISVGTSAGGAMS 158

:* :.* ***:* : : . :.*: . : * *.*** *

TanASl AIAGASGDSKLYYKYLEQIGAAMTDKNGKYISDKIDGAMAWCPITSLDQADAAYEWQMGQ 287

TanALp ALMGATGDSKKYTTYLKAIGAPLATTTGKSTSDAVAGAMAWCPITSLDTANEAYEWNMGQ 283

TanASg AIIGSSGDSSLYDDYLTEIGAVEG------VSDSVAGVMAWCPITNLDTANEAYEWNMGS 269

TanBLp ALAGASGNSAYFEPALTALGAAP-------ATDDIFAVSAYCPIHNLEHADMAYEWQFNG 222

TanBSg SLLGSTGNRAEYLSFLEEIGAELD------QRDDIFAAQCFCPITNLEHADMAYEWMFQA 212

:: *::*: : * :** * : .. .:*** .*: *: **** :

TanASl YGNEGNRKKNSFQKQLSTDLASSYASYLNKLNLKNGN-TTLSLTKSKNGQYTEGSYAKYL 346

TanALp YSNSGTRKQGTWTKALSNDMATSYAQYINKLGLKDANGKTLTLKKSTSGIYTSGTYATYL 343

TanASg TRSDLSDEE----QTISDGLATAFAKYINKLGLQDEDGNKLTLKKSDDGIYQAGSYYNYL 325

TanBLp INDWHRYQP------VAGTTKNGRPKFEPVSGQLTVEEQALSLALKAQFSTYLNQLKLTA 276

TanBSg KKIYTFNSR------VRPQIINKR-------------QQLLSQSLAAEFPEYVNSLHLDE 253

. : . *: . .

TanASl KKEIEDSATEFLNNTTFPYKQNSTEQAG-------MGNGGPSGGKPSG--KMGSMPQMRK 397

TanALp KKEVEQSLNNFLKDTTFPYKATSNEGPSGAASQTLTSGKMPSGSKPSGTAKSGSKPSGSA 403

TanASg KSVIEDSLNTFLANTTFPYDASSSSQGG------LGGGDMPTGEAPTDLGTTDDTTSIED 379

TanBLp S---DGTHLTLNEAGMGSFRDVVRQLLIS------------------------------- 302

TanBSg SLTADGRGGNFYQGILNQLSLSLNKFLAK------------------------------- 282

. : : .

TanASl QS-------------SNKTYKTMDAYLKDLNKKGTWITYDKKTKRAHITSLKDFAKYYKQ 444

TanALp PSGTATNSSS----TSGETYKTATAYIKALNKNGKWITYNAKKNTATITSVKAFVKHCKT 459

TanASg VDDINRTSSSSITIDLSGTYETAADYIAALNADSTWVTYDEDTNTASISSIADFVKYMKS 439

TanBLp ---------------SAQTAFDQGTDIHKYAG----FAVTGNQ--VTDLDLSAYLKSLT- 340

TanBSg ---------------HAQTNDEKEELARELDPQGLWCHFENGQ--ATVFDLDAYVVNYMG 325

* . .: :

TanASl PSKSVSAFDDLKRSQAENEVFGTSGSDSKLHFDQSLAKLLTENKSNYSKLNGWNSNYVSS 504

TanALp ASKDVGAFDGLTRQQTENKLFATNGS-SANHFDATISKLLTTNQSKYAKLKNYKASYAKA 518

TanASg STKSLGAFDALDLSQGENQLFGYGDG-NSVHWDSTLGDLFKG------------TDYEEA 486

TanBLp RMKAVPAFDQLDLTSPENNLFGDATA-KAKHFTA------------------------LA 375

TanBSg RKKDCPAFDSLDYQTPETEVFGNRDK-NHRHFSENVAKHIEK-------------LPALS 371

* *** * *.::*. . *: :

TanASl YKNDLTKTDKLGTSMSTRMNMYNPMYYLSDYYSGYGKSNVANHWRIRTGIQQGDTALNTE 564

TanALp YRSDLKKTDAQGSSIQKRMNLYNPLYYLTSYYDGYNTSKVAKYWRIRTGINQSDTALTVE 578

TanASg FTTDLVKTDSLGNDLTTRINMYTPLYYLTDYYGGENSSNVASYWRIRTGLSQGDTALTTE 546

TanBLp QTRSTVTAQLADAELIQA---INPLSYLTTTSS-----QVAKHWRIRHGAADRDTSFAIP 427

TanBSg DYQKAFQVDLAEEDLILARKLLNPMTFLQSDLEE---KQVASHYRICLGAKDADTSFAIS 428

. .: .: .*: :* :**.::** * : **::

TanASl TNLSLALKERVGSKNVDFKTVWDQGHTMAETSGNSDSNFIKWVESINKK-- 613

TanALp TNLALTLKQNSQVKSVDFATVWGQGHTEAERKGNNETNFIKWVNKSLK--- 626

TanASg VNLALALEN-YGVKDLDFATVWGEQHTEAEISGDSTSNFIDWVNQSLADNS 596

TanBLp IILAIMLEN--HGYGIDFALPWDIPHSGDYDLG----DLFSWIDGLCQ--- 469

TanBSg YLLALALKK--RGIDVHYELIWGMGHADADYNE----EFSQWVDAIVH--- 470

*:: *:: .:.: *. *: :: .*::

**B**

TanASl --MKKTFISLLS----ATVILSGCGVGEHQNNNSNHDAKGVN--TSNVKIKNYNQASSAL 52

TanALp -MQFRKIVPLMSGLLVMSVGLAACGHSETKTKHPTSTVAKVAKATKQTVTKADVKNAKKL 59

TanASg MPRKKWFFTSSAVLLCSAMLLTACSSSSNSSTSSSSSQNTTA---STSSLSSGEVSTTLD 57

: :.. : :: *:.*. .. ... .. . . . :.

TanASl QIDNSKWKYDSKNNVYYQLNISYVSNPQAKNVEKLGIYVPAAYFKGKKNHNGTYTVTVND 112

TanALp LINQKQWHYNATNKVYYQVGVKYGTKTTSSTYESMGIFIPAKYVNAKASGQKTYTITFNN 119

TanASg KVDNSKWQYNADDNVYYQIGISYAANPTDAEQQTLSIFVPGDYMTATDNGNGTYTCEINT 117

:::.:*:*:: ::****:.:.* ::. :.:.*::*. *.... . : *** .*

TanASl AKKVNGYSARTAPIVYPVNTPGYAEQSAPTSYRYSNISKYMKAGFIYVEAGLRGRSMSMG 172

TanALp KAKVKGYTAKTAPIVMPVNTPGYAAQTAPTGYDSS-ANKYTKAGFIYVAAGCRGLSQSDK 178

TanASg SATVGNYTSETAPIVIPINTPGYSAMSALTEYTSD-ATDYTSQGMIYVSAGLRGRDSG-- 174

.* .*::.***** *:*****: :* * * . ..* . *:*** ** ** . .

TanASl NNSSNASTKSYETGSPWGVTDLKAAIRYYRFNDSSLPGNSSKIYTFGHSGGGAQSAIAGA 232

TanALp SNGS----------SPWGVTDLKAAVRTLRLNRSRIAGNTNRVFTFGHSGGGAQSALMGA 228

TanASg --------------APSGVTDAKAAIRYLRYNQGNISGNTDSIFVFGMSGGGAQSAIIGS 220

:* **** ***:* * * . :.**:. ::.** ********: *:

TanASl SGDSKLYYKYLEQIGAAMTDKNGKYISDKIDGAMAWCPITSLDQADAAYEWQMGQYGNEG 292

TanALp TGDSKKYTTYLKAIGAPLATTTGKSTSDAVAGAMAWCPITSLDTANEAYEWNMGQYSNSG 288

TanASg SGDSSLYDDYLTEIGAVEG------VSDSVAGVMAWCPITNLDTANEAYEWNMGSTRSDL 274

:***. * ** *** ** : *.*******.** *: ****:**. ..

TanASl NRKKNSFQKQLSTDLASSYASYLNKLNLKNGN-TTLSLTKSKNGQYTEGSYAKYLKKEIE 351

TanALp TRKQGTWTKALSNDMATSYAQYINKLGLKDANGKTLTLKKSTSGIYTSGTYATYLKKEVE 348

TanASg SDEE----QTISDGLATAFAKYINKLGLQDEDGNKLTLKKSDDGIYQAGSYYNYLKSVIE 330

. :: : :* .:*:::*.*:***.*:: : ..*:*.** .* * *:* .***. :*

TanASl DSATEFLNNTTFPYKQNSTEQAG-------MGNGGPSGGKPSG--KMGSMPQMRKQS--- 399

TanALp QSLNNFLKDTTFPYKATSNEGPSGAASQTLTSGKMPSGSKPSGTAKSGSKPSGSAPSGTA 408

TanASg DSLNTFLANTTFPYDASSSSQGG------LGGGDMPTGEAPTDLGTTDDTTSIEDVDDIN 384

:* . ** :*****. .*.. . .. *:* *:. . .. .. .

TanASl ----------SNKTYKTMDAYLKDLNKKGTWITYDKKTKRAHITSLKDFAKYYKQPSKSV 449

TanALp TNSSS----TSGETYKTATAYIKALNKNGKWITYNAKKNTATITSVKAFVKHCKTASKDV 464

TanASg RTSSSSITIDLSGTYETAADYIAALNADSTWVTYDEDTNTASISSIADFVKYMKSSTKSL 444

. **:* *: ** ...*:**: ..: * *:*: *.*: * .:*.:

TanASl SAFDDLKRSQAENEVFGTSGSDSKLHFDQSLAKLLTENKSNYSKLNGWNSNYVSSYKNDL 509

TanALp GAFDGLTRQQTENKLFATNGS-SANHFDATISKLLTTNQSKYAKLKNYKASYAKAYRSDL 523

TanASg GAFDALDLSQGENQLFGYGDG-NSVHWDSTLGDLFKG------------TDYEEAFTTDL 491

.*** * .* **::*. ... . *:* ::..*:. :.* .:: .**

TanASl TKTDKLGTSMSTRMNMYNPMYYLSDYYSGYGKSNVANHWRIRTGIQQGDTALNTETNLSL 569

TanALp KKTDAQGSSIQKRMNLYNPLYYLTSYYDGYNTSKVAKYWRIRTGINQSDTALTVETNLAL 583

TanASg VKTDSLGNDLTTRINMYTPLYYLTDYYGGENSSNVASYWRIRTGLSQGDTALTTEVNLAL 551

*** *..: .*:*:*.*:***:.**.* ..*:**.:******:.*.****..*.**:*

TanASl ALKERVGSKNVDFKTVWDQGHTMAETSGNSDSNFIKWVESINKK-- 613

TanALp TLKQNSQVKSVDFATVWGQGHTEAERKGNNETNFIKWVNKSLK--- 626

TanASg ALEN-YGVKDLDFATVWGEQHTEAEISGDSTSNFIDWVNQSLADNS 596

:*:: *.:** ***.: ** ** .*:. :***.**:.

**C**

TanBLp -MSNRLIFDADWLVPEQVQVAGQAIQYYAARNIQYVQHPVAAIQVLNVFVPAAYLHGSSV 59

TanBSg MSINQWIFDETNNCYMSLKNVYCAQPKDSELEALHIFVPAVYMTADGTIDRDAVVTNKNG 60

*: *** .:: . * : : :: *.. : . ..: * : ...

TanBLp NGYQRATAPILMPNTVGGYLPGPADDPQRVTWPTNAGTIQQALKRGYVVVAAGIRGRTTV 119

TanBSg TIYTSQTVPIIFYNDIGGYAECQP--------AMVTPRNQRYLEDGYVLVSVGARGR--- 109

. * *.**:: * :*** . . : *: *: ***:*:.* ***

TanBLp DKSGQRVGQAPAFIVDMKAAIRYVKYNQGRLPGDANRIITNGTSAGGATSALAGASGNSA 179

TanBSg -QSQNGIGKAPAGLVDLKAAVRWLRKHHNDIPGDIEKIISVGTSAGGAMSSLLGSTGNRA 168

:* : :*:*** :**:***:*::: ::. :*** ::**: ******* *:* *::** *

TanBLp YFEPALTALGAAP-ATDDIFAVSAYCPIHNLEHADMAYEWQFNGINDWHRYQPVAGTTKN 238

TanBSg EYLSFLEEIGAELDQRDDIFAAQCFCPITNLEHADMAYEWMFQAKKIYTFNSRVRPQIIN 228

: . * :** *****...:*** *********** *:. : : . * *

TanBLp GRPKFEPVSGQLTVEEQALSLALKAQFSTYLNQLKLTAS---DGTHLTLNEAGMGSFRDV 295

TanBSg KR-------------QQLLSQSLAAEFPEYVNSLHLDESLTADGRGGNFYQGILNQLSLS 275

* :* ** :* *:*. *:*.*:* * ** .: :. :..:

TanBLp VRQLLISSAQTAFDQGTDIHKYAG----FAVTGNQVTDLDLSAYLKSLT-RMKAVPAFDQ 350

TanBSg LNKFLAKHAQTNDEKEELARELDPQGLWCHFENGQATVFDLDAYVVNYMGRKKDCPAFDS 335

:.::* . *** :: :: . ..*.* :**.**: . * * ****.

TanBLp LDLTSPENNLFGDATAKAKHFTA-----------LAQTRSTVTAQLADAELIQA---INP 396

TanBSg LDYQTPETEVFGNRDKNHRHFSENVAKHIEKLPALSDYQKAFQVDLAEEDLILARKLLNP 395

** :**.::**: : :**: *:: :.:. .:**: :** * :**

TanBLp LSYLTTTSS--QVAKHWRIRHGAADRDTSFAIPIILAIMLENHGYGIDFALPWDIPHSGD 454

TanBSg MTFLQSDLEEKQVASHYRICLGAKDADTSFAISYLLALALKKRGIDVHYELIWGMGHADA 455

:::* : . ***.*:** ** * ******. :**: *:::* .:.: * *.: *:.

TanBLp YDLGDLFSWIDGLCQ 469

TanBSg DYNEEFSQWVDAIVH 470

:: .*:*.: :

**Additional file 1: Figure S1. Comparison of amino acid sequences of bacterial tannases**. (A) TanA_Sl_ from *Staphylococcus lugdunensis*, TanA_Lp_ and TanB_Lp_ from *Lactobacillus plantarum*, and TanA_Sg_ and TanB_Sg_ from *Streptococcus gallolyticus* subsp. *gallolyticus*. (B) Alignment of TanA or (C) TanB proteins. Multiple alignments were done using the program ClustalW2 after retrieval of sequences from BLAST homology searches. Residues that are identical (*), conserved (:) or semiconserved (.) in all sequences are indicated. Dashes indicated gaps introduced to maximize similarities. The vertical line indicated the predicted peptide signal cleavage site. The serine hydrolase conserved motif is highlighted in yellow; residues of the catalytic triad identified in the structure of TanB_Lp_ are highlighted in blue; and residues which make contacts with the three hydroxyl groups of gallic acid are highlighted in pink color.
