# Supplementary material for: Genetic and biochemical approaches towards unravelling the degradation of gallotannins by Streptococcus gallolyticus
Source: Microb Cell Fact. 2014 Oct 31;13:154. doi: 10.1186/s12934-014-0154-8 (PMC4218992; doi:10.1186/s12934-014-0154-8)
Supplement: Additional file 2: Table S1. — Characteristics of S. gallolyticus tannases. [file 12934_2014_154_MOESM2_ESM.doc]

**Table S1. Characteristics of *S. gallolyticus* tannases**

|  | **TanA_Sg_** | **TanB_Sg_** |
| --- | --- | --- |
| Signal peptide | Yes | Non |
| Molecular mass (mature protein) | 60.7 kDa | 52.9 kDa |
| Isoelectric point (*p*I) | 3.9 | 5.1 |
| Specific activity | 256 U/mg | 577 U/mg |
| Temperature (optimal) | 37 ºC | 45 ºC |
| pH (optimal) | 6.0 | 7.0 |
| Substrate range | Gallate and protocatechuate esters | Gallate and protocatechuate esters |
| Gene induction (by methyl gallate) | Yes (14-fold) | Yes (6-fold) |
| Activators | Ca^2+^, K^+^, Tween-80 | Tween-80, Ca^2+^, K^+^ |
| Inhibitors | Hg^2+^, Zn^2+^, Mg^2+^,  EDTA, βME^a^ | Hg^2+^, Zn^2+^, urea,  βME^a^ |

^a^, β-mercaptoethanol
