# Supplementary material for: Genetic and biochemical approaches towards unravelling the degradation of gallotannins by Streptococcus gallolyticus
Source: Microb Cell Fact. 2014 Oct 31;13:154. doi: 10.1186/s12934-014-0154-8 (PMC4218992; doi:10.1186/s12934-014-0154-8)
Supplement: Additional file 3: Table S2. — Primers used for PCR and qPCR analysis. [file 12934_2014_154_MOESM3_ESM.doc]

**Table S1. Primers used for PCR and qPCR analysis**

| **Target gene** | Primer name | **Sequence 5´→3^b^** | **Amplicon size (bp)** |
| --- | --- | --- | --- |
| *tanA_Sg_* | 803 | *GGTGAAAACCTGTATTTCCAGGGC*tctagtacctctagctccagctcgc^a^ | 1750 |
|  | 804 | *ATCGATAAGCTTAGTTAGCTAtta*tgagttgtcagctaagctttgatta |  |
| *tanB_Sg_* | 774 | *TAACTTTAAGAAGGAGATATACATatg*tcgattaatcaatggatttttg | 1465 |
|  | 775 | *GCTATTAATGATGATGATGATGATGATG*aacaatggcatccacccattg |  |
| *tanA_Sg_* | 1552 | TGGCACTTGCCCTTGAAAA | 58 |
|  | 1553 | CCCCAAACCGTTGCAAAA |  |
| *GALLO_1608* | 1554 | GCGATTGGTGGTGTCTTACTTG | 64 |
|  | 1555 | GACCTTGTCCACCCATGAGGTA |  |
| *tanB_Sg_* | 1556 | CTTGCAGTCTGACTTGGAGGAA | 65 |
|  | 1557 | CTTTTGCCCCTAAACAAATACGA |  |
| *GALLO_1610* | 1558 | AGCCAATCCAAATGCCAAATA | 63 |
|  | 1559 | GAAAGAGTTGGGAGTGTGATGACTT |  |
| *sgdD* | 1560 | ATTGCTGGCGCTTTTGAAA | 65 |
|  | 1561 | TCAATTTTTAACCATGTGCCAATC |  |
| *sgdB* | 1562 | CGCCTGTACCAGCCTTTTACA | 76 |
|  | 1563 | TCTAGTAATTTAGCAGTATTGTGGTCGAT |  |
| *sgdC* | 1564 | CCACCCACTTGACCCATCAG | 60 |
|  | 1565 | CCTCGTACACGGATGTGTTCTG |  |
| *GALLO_1614* | 1566 | GTCGTCCGTTGGAGGTTGAT | 65 |
|  | 1567 | TGTTGTTTTCTTCTTACGACGGATT |  |
| *16S rRNA* | 598 | CTGCTGCCTCCCGTAGGA | 57 |
|  | 1576 | GGGTGATCGGCCACACTG |  |

^a^ The nucleotides pairing the expression vector sequence are indicated in italics, and the nucleotides pairing the *tanA_Sg_* and *tanB_Sg_* gene sequences are written in lowercase letters
